# Statistical Issues in TBI Clinical Studies

**DOI:** 10.3389/fneur.2013.00177

**Published:** 2013-11-19

**Authors:** Paul E. Rapp, Christopher J. Cellucci, David O. Keyser, Adele M. K. Gilpin, David M. Darmon

**Affiliations:** ^1^Department of Military and Emergency Medicine, Uniformed Services University, Bethesda, MD, USA; ^2^Aquinas, LLC, Berwyn, PA, USA; ^3^Albertus Magnus Foundation, Berwyn, PA, USA; ^4^Arnold and Porter, LLP, Washington, DC, USA; ^5^Department of Epidemiology and Preventive Medicine, University of Maryland, College Park, MD, USA; ^6^Department of Mathematics, University of Maryland, College Park, MD, USA

**Keywords:** neuropsychiatric diagnosis, statistical errors, research design, Mahalanobis distance, statistical variability, treatment effects

## Abstract

The identification and longitudinal assessment of traumatic brain injury presents several challenges. Because these injuries can have subtle effects, efforts to find quantitative physiological measures that can be used to characterize traumatic brain injury are receiving increased attention. The results of this research must be considered with care. Six reasons for cautious assessment are outlined in this paper. None of the issues raised here are new. They are standard elements in the technical literature that describes the mathematical analysis of clinical data. The purpose of this paper is to draw attention to these issues because they need to be considered when clinicians evaluate the usefulness of this research. In some instances these points are demonstrated by simulation studies of diagnostic processes. We take as an additional objective the explicit presentation of the mathematical methods used to reach these conclusions. This material is in the appendices. The following points are made: (1) A statistically significant separation of a clinical population from a control population does not ensure a successful diagnostic procedure. (2) Adding more variables to a diagnostic discrimination can, in some instances, actually reduce classification accuracy. (3) A high sensitivity and specificity in a TBI versus control population classification does not ensure diagnostic successes when the method is applied in a more general neuropsychiatric population. (4) Evaluation of treatment effectiveness must recognize that high variability is a pronounced characteristic of an injured central nervous system and that results can be confounded by either disease progression or spontaneous recovery. A large pre-treatment versus post-treatment effect size does not, of itself, establish a successful treatment. (5) A procedure for discriminating between treatment responders and non-responders requires, minimally, a two phase investigation. This procedure must include a mechanism to discriminate between treatment responders, placebo responders, and spontaneous recovery. (6) A search for prodromes of neuropsychiatric disorders following traumatic brain injury can be implemented with these procedures.

## Introduction

We consider here statistical issues that are associated with four processes encountered in clinical studies. They are diagnosis, longitudinal assessment of treatment, evaluation of treatment effectiveness, and the identification of prodromes of psychiatric illness. An emphasis is placed on traumatic brain injury, but the conclusions generalize to other disorders. In mathematical terms diagnosis is a classification process. In diagnosis we ask: given a specific patient and a set of measurements obtained from that individual, what is the probability of that individual’s membership in previously identified and characterized populations, including a group of appropriately matched healthy controls? At present the specification of clinical populations follows conventional diagnostic structures, major depressive disorder, PTSD, schizophrenia, and the like. Neuropsychiatric diagnosis is now undergoing a reassessment (1–3). We want to make an essential point. Though diagnostic criteria may change, the statistical issues that must be addressed in their implementation remain the same.

Longitudinal assessment and the evaluation of treatment effectiveness is a classification problem in the limited sense that it involves calculations of the probability that the patient is a member of an appropriately matched healthy control group, which should increase longitudinally, and calculation of the probability that the patient is a member of the clinical group identified in diagnosis, which should decrease during the course of treatment. The calculation of these membership probabilities provides a global assessment, but assessment of treatment adherence, consistency of treatment, inter-rater reliability, and examination of appropriately constructed controls arms must complement these calculations. Some of these issues are considered in the nine questions addressed in Section “Evaluation of Treatment Effectiveness must Recognize that High Variability is a Pronounced Characteristic of an Injured Central Nervous System and that Results can be Confounded by Either Disease Progression or Spontaneous Recovery. A Large Pre-Treatment Versus Post-Treatment Effect Size does not of Itself Establish a Successful Treatment.”

The statistical implementation of diagnosis and the statistical assessment of treatment effectiveness have important differences. In principle, the assessment of treatment effectiveness can be made with a single calculation: probability of membership in the control group. This can be done in the absence of a diagnosis. A statistically based diagnosis is based on the maximum membership probability determined across a large number of clinical groups. The diagnostic process can fail if the measures lack between group-specificity. Longitudinal assessment can fail if the measures have low test-retest reliability. The operational difficulties of this approach to diagnosis and treatment evaluation should not be underestimated.

As a specific example, we will present this discussion in terms of classification between a control population and a TBI population where it is to be understood that this is done without prejudice as to the defining specification of the clinical group and without returning to a discussion of the logical validity of treating TBI as a diagnostic category (4). As noted in the abstract, we recognize that none of the ideas presented here are new. Our purpose is to state them, to support them with simulations of diagnostic processes, and to present concisely the essential mathematical material in appendices.

## A Statistically Significant Separation of a Clinical Population from a Control Population Does Not Ensure a Successful Diagnostic Procedure

We consider here the simplest case, a two group discrimination between-Group A, the control population, and Group B, the TBI population. A collection of diagnostic measures is taken from each participant. Candidate measures include plasma and CSF biomarkers, results from neuropsychological evaluations, measures of autonomic nervous system function derived from heart rate variability assessments, quantitative EEG measures, measures of cognitive event related potentials, eye tracking results, and balance studies. For the present analysis we assume that the measures are continuous variables. It is possible to generalize the analysis to incorporate nominal and ordinal variables (5). The same qualitative conclusions are found in this expanded analysis.

The first question to be addressed is: using this set of measures can we show that Group A (Control) is different from Group B (TBI) and what is our confidence in that separation? This is most commonly accomplished by calculating *P*_SAME_ (*G*_A_, G_B_) with a multivariate *F*-test. It is generally supposed that a small value of *P*_SAME_ indicates that the two groups are not the same. While this is usually an operationally valid interpretation, it is not strictly speaking correct. A small value of *p* does not prove that two groups are not the same but rather that it is unlikely that they are the same. The usual misinterpretation of *p*-values is that a large *p* value (especially a value near one) is evidence for the null hypothesis. This isn’t the case since under the null hypothesis all *p*-values are equally likely (under the null they’re uniformly distributed). This is why we can use a small value of *p* to reject the null hypothesis, but we cannot use a large value to accept the null hypothesis. Murdoch et al. (6) stress that *p*-values are random variables. As a general observation *p*-values should only be used as evidence against a null hypothesis. The details of the calculation and a technical statement of the interpretation of *P*_SAME_ (*G*_A_, *G*_B_) are given in Section “Calculation of *P*_SAME_ (*G*_A_, *G*_B_)” in Appendix.

A more serious misinterpretation of *P*_SAME_ is encountered when it is suggested that a small value of *P*_SAME_ as determined in an *F*-test demonstrates that the measures used in the calculation can be used to diagnose TBI. This is not the case. Let *P*_ERROR_ (*G*_A_, *G*_B_) be the error rate observed when the measures are used to classify a specific individual between Group A and Group B. A procedure for establishing a theoretical estimate of assignment error, *P*_ERROR − FORMULA_ is given in Section “Calculation of *P*_ERROR − FORMULA_ (*G*_A_, *G*_B_)” in Appendix. We stress that the theoretical *P*_ERROR − FORMULA_ can be a serious underestimate of the true error rate, *P*_ERROR − EMPIRICAL_. This issue is addressed presently.

**Figure 1:**
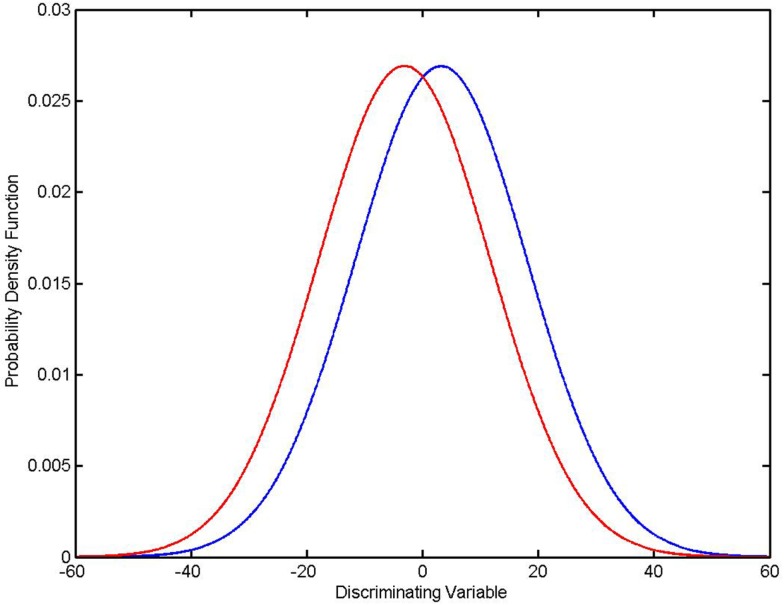
***P*_SAME_ ≠ *P*_ERROR_**. Two normal distributions: μ_A_ = 3.2117, σ_A_ = 14.8328 (in blue), μ_B_ = − 3.1433, (in red) σ_B_ = 14.8255, *N*_A_ = *N*_B_ = 500. Given assumptions that the distributions are normal and that an optimal Bayesian classifier is used to classify individual elements, *P*_SAME_ = 2.1096 × 10^−11^ and *P*_ERROR − FORMULA_ = 0.4078.

Simply put, *P*_SAME_ ≠ *P*_ERROR_, and in some cases *P*_ERROR_ ≫ *P*_SAME_. An example is shown in the diagram (Figure 1). In this case there was a single discriminating variable. Two normal distributions were generated computationally, where *N*_A_ and *N*_B_, the number of members in each group, is 500 for both distributions. The mean values and standard deviations of the two distributions were μ_A_ = 3.2117, σ_A_ = 14.8328, μ_B_ = −3.1433, and σ_B_ = 14.8255. Using the formulas given in the appendices it was found that *P*_SAME_ = 2.1096 × 10^−11^ while *P*_ERROR − FORMULA_ = 0.4078. It should be remembered that the expected error rate in a random assignment between two groups is 0.5. Thus the classifier is performing only marginally better than random assignment even though *P*_SAME_ ≈ 10^−11^.

Theoretical classification error rates must be considered with care. The formula for *P*_ERROR_ can give a serious underestimate of the true classification error rate. Empirically determined error rates give a better test of diagnostic reliability. A discussion of empirical estimates of classification error must be preceded by a specification of the procedure used to classify individual participants between-groups. Three methods that can be used with continuous variables are presented in Section “Three Classifiers for Continuous Measures” in Appendix: classification by minimum Mahalanobis distance, classification by maximum Bayesian likelihood, and classification with a quadratic classifier, where it is shown that classification by maximum Bayesian likelihood is equivalent to classification by a quadratic classifier.

Given these classification criteria, it is possible to construct systematic empirical procedures for estimating classification error rates [(7, 8) Chapter 22, Section 8; (5) Chapter 7, Section 10). As previously noted the equation for *P*_ERROR − FORMULA_ (*G*_A_, *G*_B_) given in Section “Calculation of *P*_ERROR − FORMULA_ (*G*_A_, *G*_B_)” in Appendix is the best available estimate of dichotomous classification error when only group means and covariances are available, but it can seriously underestimate true error rates. The *k*-fold cross validation and the out-of-sample validation tests provide an empirical estimate.

There is a distinction between the *k*-fold cross validation and an out-of-sample validation (8). This technical distinction is presented in Section “Simulation Studies Comparing *P*_ERROR − EMPIRICAL_ and *P*_ERROR − FORMULA_” in Appendix. The essential point is the following: in both the *k*-fold cross validation and the out-of-sample validation, the elements to be classified are not used in the construction of the classifier. This is critical to the validity of the assessment. Within-sample testing, where an element that is classified is also used in the construction of the classifier, can give a serious underestimate of the true error rate. This is especially likely to occur if group population numbers are low. Wasserman [(8), p. 363] gives an example that emphasizes this distinction. Similarly, Watanabe et al. (9) have published an example comparing *P*_ERROR − FORMULA_ calculated from the equation and four different empirical determinations of classification error. Two of the empirical determinations inappropriately used the element to be classified in the classifier. They gave artifactually low error rates (7.7 and 0%). The legitimate empirical classification that did not use the elements that were classified in the classifier gave much higher error rates (85 and 69%). The theoretical *P*_ERROR − FORMULA_ calculated using the formula in Section “Calculation of *P*_ERROR − FORMULA_ (*G*_A_, *G*_B_)” in Appendix was 15.7%.

We wish to draw attention to the difference in the error rate predicted using the previously presented formula, *P*_ERROR − FORMULA_, and error rate determined in *k*-fold calculations, *P*_ERROR − EMPIRICAL_. While *P*_ERROR − FORMULA_ = 0.157, *P*_ERROR − EMPIRICAL_ varies between 0.46 and 0.85 (see Table 1 above). *P*_ERROR − FORMULA_ does, however, have a great advantage. It’s easy to calculate. This is especially true of univariate discriminations. In the case of a single variable classifier, *P*_ERROR − FORMULA_ can be calculated with just means and standard deviations. These data are typically included in published reports. In contrast, calculations of *P*_ERROR − EMPIRICAL_ require access to the full participant-by-participant data set. If it can be shown that the more reliable *P*_ERROR − EMPIRICAL_ is always greater than or approximately equal to the readily calculated *P*_ERROR − FORMULA_, then a large value of *P*_ERROR − FORMULA_ calculated using publically accessible published results can be used to dismiss spurious claims of candidate classifiers. This possibility leads to the following motivating question. Is *P*_ERROR − EMPIRICAL_ always greater than or approximately equal to *P*_ERROR − FORMULA_, where *P*_ERROR − EMPIRICAL_ is determined by a *k*-fold cross validation? This question is addressed in the simulation studies presented in Section “Simulation Studies Comparing *P*_ERROR − EMPIRICAL_ and *P*_ERROR − FORMULA_” in Appendix. The calculations reported there suggest that the reliable empirically determined classification error rate is either approximately equal to or greater than the easily calculated formula-based estimate of classification error. It follows that calculations of *P*_ERROR − FORMULA_ using published values of means and standard deviations can effectively challenge claims of effective diagnostic classification. Operationally, if the easily calculated *P*_ERROR − FORMULA_ is large, then an effective classification will most probably be impossible.

**Table 1:** **EEG classification error rates**.

Condition	Error rate of random assignment (%)	Error rate minimum Mahalanobis distance within-sample classification (%)	Error rate maximum Bayesian likelihood within-sample classification (%)	Error rate minimum Mahalanobis distance *k*-fold classification (%)	Error rate maximum Bayesian likelihood *k*-fold classification (%)
Eyes open	50	7.7	0	85	69
Eyes closed	50	0	0	46	46

The formula determined error rate is 15.7%, a serious underestimate of the true error rate. When the element to be classified is used in the construction of the classifier, this is the within sample error rate, the calculated error rate is again significantly smaller than the error rate determined by a *k*-fold classification. *k*-fold classification tests provide a test of classifier performance in actual practice [Modified from Watanabe et al. (9)].

## Adding More Variables to a Diagnostic Discrimination Can, in Some Instances, Actually Reduce Classification Accuracy

It is commonly supposed that adding a variable to a multivariate classifier will improve classification performance. In our context, it is supposed that adding a clinical measure will improve diagnostic accuracy. Is this indeed the case? The theoretical and practical answers to this question are different. Theoretically, if all variables are known, that is means and covariances are known exactly, then adding a variable will not degrade the classifier. There is one qualification to this theoretical statement. If two variables are exactly correlated, then the covariance matrix is singular. Inverting the covariance matrix, which is required to calculate the Mahalanobis distance, is impossible and the classification fails.

The practical answer to the question “Can adding a variable hurt?” is more complex. If the added variable is highly, but not exactly, correlated with a variable already in the discrimination, then the covariance matrix is near-singular. Inverting the near-singular matrix introduces numerical errors that can actually result in worse classification performance. A second potential problem created by introducing a large number of variables is the creation of false correlations. This is analogous to over-fitting a model. Examples are given in Hastie et al. [(5), pp. 245 and 247). Including all available measures is, therefore, not necessarily the best course.

**Figure 2:**
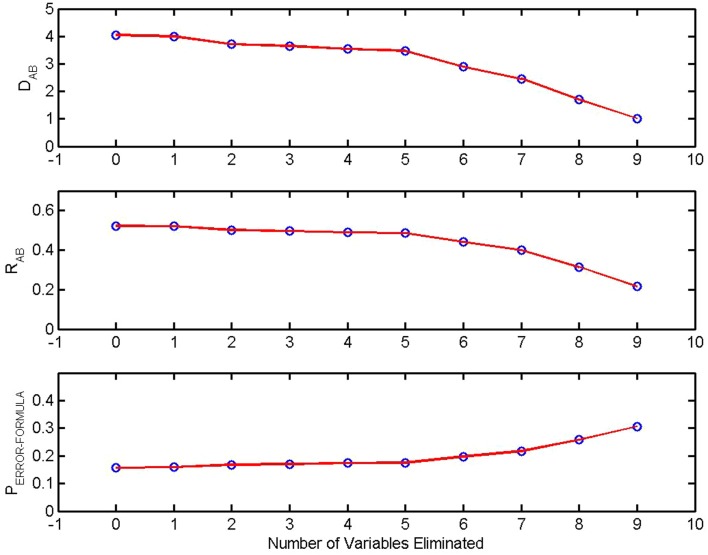
**Sensitivity of discrimination and backward elimination: The between-group Mahalanobis distance *D*_A,B_, the coefficient of determination *R*_A,B_, and the theoretical probability of error in a pairwise classification, *P*_ERROR − FORMULA_ are plotted as a function of the number of measures eliminated from the discrimination**. At each step the least significant variable was removed. From Watanabe et al. (9).

Backward elimination is based on *R*_A,B_, the coefficient of determination between-Group A and Group B. It is the fraction of total between-group variance that can be accounted for with a given set of measures [(10), p. 96, see “Coefficient of Determination” in Appendix). The example of backward elimination presented here (Figure 2) is from the previously described study of Watanabe et al. (9). In this study multichannel EEGs were obtained in two conditions, eyes closed, no task, and eyes open, no task. Ten measures calculated from each multichannel signal were used to construct the first version of the classifier. *R*_A,B_, *D*_A,B_, the between-group Mahalanobis distance and the theoretical *P*_ERROR − FORMULA_ were calculated using all 10 variables. The coefficient of determination was then calculated using the 10 possible combinations of 9 variables. The variable that made the smallest contribution to the coefficient of determination (equivalently the smallest contribution to the Mahalanobis distance) was eliminated from the discrimination. The process was repeated sequentially. With each iteration the variable making the smallest contribution to the variance is removed. The effect on *R*_A,B_, *D*_A,B_, and *P*_ERROR − FORMULA_ is shown in the diagram. As would be expected *R*_A,B_ and *D*_A,B_ decrease and *P*_ERROR − FORMULA_ increases as variables are eliminated.

**Figure 3:**
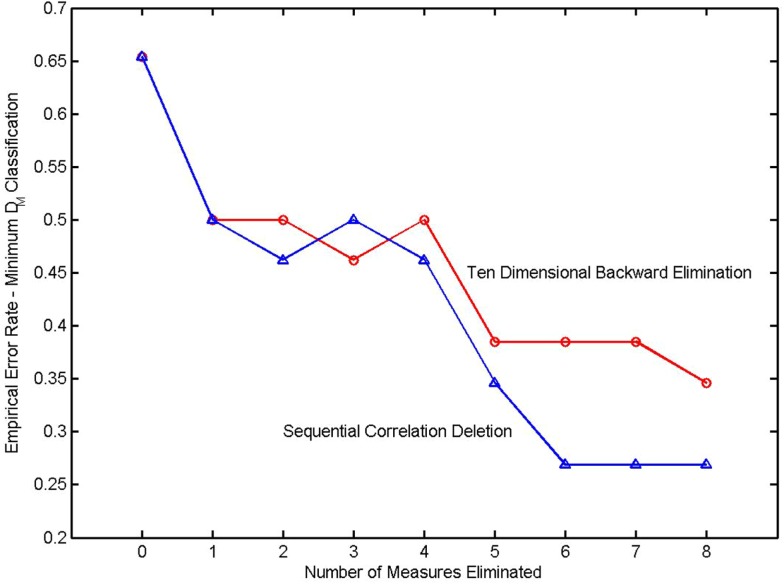
**Error rate in a *k*-fold cross validation as a function of the number of variables eliminated from the classifier**. The elimination sequence in the upper trace (denoted by circles) was determined by a backward elimination. The elimination sequence of the lower trace (triangles) was determined in a sequential correlation deletion. From Watanabe et al. (9).

It might be supposed that the empirically determined classification error rate, where classification is based on the minimum Mahalanobis distance, would also increase as variables are eliminated. The error rate of an *N*-fold cross validation is shown in the next diagram (Figure 3). It is seen that the error rate actually decreases as variables are eliminated in a backward elimination. The discriminating measures are highly correlated. The covariance matrix is extremely ill-conditioned. Inverting the matrix causes numerical errors that more than equal any discriminatory power that might be conferred by the addition of a highly correlated covariate.

The results in this diagram were obtained using the LU decomposition to invert the covariance matrix. The LU decomposition is a generically applicable procedure for inverting a matrix. It does not exploit the structure of a covariance matrix (positive semidefinite and symmetric). An inversion procedure utilizing these properties was derived in Watanabe et al. (9) and was also applied to this classifier. Due to the very high correlations between measures, there was no significant improvement.

A second model selection procedure, sequential correlation deletion (9), was used with the same data, and the results are also shown in the diagram. The process began by observing high correlations between complexity and redundancy measures. Three redundancy measures were eliminated and the corresponding complexity measures were retained. The process continued by retaining measures that had a high coefficient of determination and eliminating measures that were highly correlated but had smaller coefficients of determination. This procedure was unsystematic but nonetheless more effective in reducing the empirical *N*-fold classification error.

## A High Sensitivity and Specificity in a TBI Versus Control Population Classification Does Not Ensure Diagnostic Success When the Method is Applied in a More General Neuropsychiatric Population

Sensitivity and specificity are regarded as being the dispositive measures of a diagnostic process. Quantitative definitions are given in Section “Sensitivity and Specificity” in Appendix. Stated qualitatively, sensitivity is the test’s ability to correctly detect a condition when it is present. Specificity is the test’s ability to give a negative result when the condition is absent. In the present context, high sensitivity means that if a patient has sustained a TBI, he or she will be identified as TBI positive. A high value of specificity indicates that the test result will be negative if the patient did not sustain a TBI.

Reports of high specificity in the identification of TBI must be interpreted with care. This is particularly true if the assessment is based on psychophysiological measures (heart rate variability, quantitative EEG, event related potentials, eye tracking). A multivariate diagnostic procedure may have a high value of specificity in a carefully constructed clinical study that included a group of healthy controls and a group of TBI patients selected to exclude comorbid neuropsychiatric conditions. The test’s specificity might well be lost in practical applications assessing a less restricted patient population. Measures of EEG/MEG coherence and synchronization provide an instructive example. These measures can be altered following a TBI (11, 12), but they can also be altered in other disorders. General reviews of coherence and synchronization changes in neuropsychiatric disorders are given in Herrmann and Demiralp (13), Schnitzler and Gross (14), and Uhlhaas and Singer (15). Specific examples include AD/HD (16), alcohol abuse (17), alexithymia (18), autism (19), bipolar disorders (20), dementia (17, 21), hallucinations (22), HIV dementia (23), migraine (24), multiple sclerosis (17), Parkinson’s disease (25), PTSD (26, 27), and schizophrenia (28). Similar indications of a lack of specificity can be observed with other psychophysiological measures. Heart rate variability is altered in anxiety (29), chronic fatigue syndrome (30), depression (31), pain (32), panic disorder (33), Parkinson’s disease (34), PTSD (35), schizophrenia (36), and TBI (37). These citations are representative examples drawn from a larger literature. Small world models can be used to quantify CNS functional connectivity revealed by MEG and high density EEG recordings. Altered small world parameters are seen following traumatic brain injury (38, 39), but also in schizophrenia (40), dementia of Alzheimer’s type (41), epilepsy (42), and in patients with CNS tumors (43). Thus, as in previous examples, small world measures are sensitive to CNS pathology but are non-specific.

If an assessment of a typical neuropsychiatric population is based on psychophysiological measures, specificity will probably be lost. It is possible, however, that a good statistical separation could be obtained between patients and controls. Given our present understanding we may be able to establish that something is wrong because the probability of membership in the control group is low, but we can’t say what is wrong because we can’t discriminate between TBI, depression or bipolar disorder. Therefore the report of high control/TBI specificity in a clinical study can be of limited utility in clinical practice.

## Evaluation of Treatment Effectiveness Must Recognize That High Variability is a Pronounced Characteristic of an Injured Central Nervous System and That Results Can be Confounded by Either Disease Progression or Spontaneous Recovery. A Large Pre-Treatment Versus Post-Treatment Effect Size Does Not of Itself Establish a Successful Treatment

When considering the responses to treatment a distinction must be made between the evaluation of between-group differences and the evaluation of changes within a given individual. We consider first between-group assessments. Evaluation of group responses to treatment raise several challenges that are particularly severe in the case of traumatic brain injury. Consider the simplest case. As before, it is supposed that a set of measures is obtained from all participants. Using the procedures outlined in Section “Calculation of *P*_SAME_ (*G*_A_, *G*_B_)” in Appendix it is possible to compute the between-group distances between sets of measure vectors. The distance between the pre- and post-treatment measures should increase in response to treatment, and the separation between the TBI population and the healthy control population should decrease in response to treatment. The classical measure of treatment, the effect size, quantifies between-group separation for the special case of a single outcome measure. Three commonly employed measures of effect size, Cohen’s *d*, Glass’s Δ, and Hedge’s *g*, are presented in Section “Calculation of Single-Variable Effect Size” in Appendix where it is seen that Hedges’ *g* is the Mahalanobis distance for *Z* = 1. But is this enough? In the case of traumatic brain injury studies, straight forward measurement of pre- to post-treatment effect size and its multivariate generalizations is often not adequate. Limitations encountered when effect size is the sole metric of treatment response are considered at the end of this section. Several complicating issues need to be considered first: high intra-individual longitudinal variation, continued disease progression and spontaneous recovery.

### Intra-individual variability

A high degree of variability is a long known characteristic of an injured central nervous system [(44) reprinted 1958, (45)]. Results from longitudinal neuropsychological testing of traumatic brain injury patients provide quantitative examples. In a study with 12 participants (six patients and six controls), Bleiberg et al. (46) measured within-day and across-day neuropsychological performance. Tests were administered 30 times over 4 days. Control subjects showed consistent improvement due to learning effects. Patients showed “erratic and inconsistent performance.” The patients presented mild to moderate TBI at the time of injury. They were 12–30 months post-injury and all had made an excellent recovery as evidenced by a return to pre-injury vocational and social status. Bleiberg et al. report, however, that “Inconsistent performance was observed even in those subjects with TBI whose initial performance was equal to or better than that of control subjects.” Similarly, Cole et al. (47) conducted a test-retest reliability study of four neurocognitive assessment tools: Automated Neuropsychological Assessment Metrics (ANAM4), CNS-Vital Signs, CogState, and Immediate Post-Concussion Assessment and Cognitive Test (ImPACT). Participants deemed to have inadequate effort during one or both testing sessions, as assessed by the instrument’s scoring algorithm, were removed from the analysis. Test-retest reliability was quantified with the intraclass correlation coefficient. Cole et al. concluded that the test-retest reliability of all four tools was “lower than desired for clinical decision making.” Several factors can contribute to this variability. One is the previously mentioned intrinsic variability of the injured central nervous system. Failure to make an adequate effort is also frequently cited (48). Inadequate effort may be of neurological origin and may be intermittent, or it may be the result of malingering. There is a substantial literature describing procedures to detect malingering in neuropsychological testing (49–52) that can be applied to this analysis. Whatever the cause, the complications of high intra-individual variability must not be ignored.

### Disease progression

TBI patients can, in some instances, experience continuing deterioration over an extended post-injury period. Diffuse axonal injury following traumatic brain injury provides a pertinent example. Diffuse axonal injury is TBI-induced scattered destruction of white matter tracts. It was first described by Lindenberg et al. (53) and by Strich (54). Disconnection of axons at the time of injury (primary axotomy) is relatively rare (55). More typically, diffuse axonal injury is a progressive process that develops after injury (56–60). In rats, progressive loss of brain tissue and deterioration of cognitive performance can continue for a year following injury (58, 61, 62). As a cautionary observation, Maxwell et al. (63) note that animal models do not reproduce exactly the time course of injury that occurs in humans. Nonetheless, they conclude that “axonal change is, probably, more widespread and occurs over a longer post-traumatic time in the injured brain than had previously been appreciated.” The observations in animal models are consistent with human studies that show progressive radiological alteration following traumatic brain injury (64–68). These results are also consistent with clinical experience which has identified delayed onset neuropsychiatric disorders following traumatic brain injury. These disorders include psychosis (69–71), depression (72–76), and post-traumatic stress disorder (72, 73, 77, 78). It follows that the possibility of progressive post-injury deterioration must be incorporated in the statistical design of clinical studies of traumatic brain injury.

### Spontaneous recovery

Conversely, other patients may present a recovery that would have occurred in the absence of treatment. Spontaneous recovery often occurs following mild traumatic brain injury (79) and is also commonly observed in other neuropsychiatric disorders, for example depression. Posternak and Miller (80) conducted a meta-analysis of the course of untreated depression using studies that included a waitlist control group. In the short term (2–20 weeks) depressive symptoms decreased by 10–15% without treatment, and approximately 20% of untreated participants presented a spontaneous remission. In a subsequent study, Posternak et al. (81) found a lower limit median duration of untreated depression of approximately 23 weeks.

To summarize, high intrinsic variability and the potential for significant post-injury deterioration or spontaneous recovery during a clinical trial place exceptional demands for statistical safeguards when working with this patient population. Several procedures for assessing change have been proposed. The Reliable Change Index (82) determines the statistical significance of change based on a comparison of the difference between initial and retest scores obtained from a reference group. Chelune et al. (83) published a variant of the Reliable Change Index that includes a correction for practice effects which is particularly important if the results of neuropsychological tests are being used as outcome measures. This correction, however, assumes that individuals will present the same practice effect irrespective of the initial score (84). The simple regression model of McSweeny et al. (85) endeavors to correct for both practice effects and regression to the mean. A multiple regression model (86) incorporates additional factors such as age education and intellectual ability. We require a statistical procedure that incorporates elements from these earlier methods and generalizes them to incorporate data from longitudinal control groups.

We consider here the development of statistical procedures for conducting an investigation of a single form of treatment and defer consideration of more complicated comparative studies with multiple treatment arms. Increased confidence in the results will be obtained if the design includes a healthy control group and a waitlist control group that meets the same inclusion/exclusion criteria as the treatment group. As will be shown, data from the waitlist group will be used to quantify changes due to spontaneous recovery or continued disease progression that can occur in the absence of treatment. Data from the healthy control group provide a specification of treatment objectives. The greatest simplicity of interpretation is obtained if all participants are assessed at two time points, at an initial T_I_ prior to treatment and a final T_F_ following treatment. The time interval between initial and final measurements should be the same for the treatment group and for the two control groups. The second measurement for the healthy control group, which is presumably clinically stable during this interval, is valuable because familiarization with the assessment procedure, for example familiarization with an EEG lab, can affect psychophysiological results and practice effects can distort the results of neuropsychological tests. An expanded design can include a placebo control group. Placebo controls are considered in the next section. There are six sets of measure vectors.

G_TI_ the set of measure vectors obtained from the treatment group at the initiation of treatment,
G_TF_ the set of measure vectors obtained from the treatment group at the conclusion of treatment,
G_HI_ the set of measure vectors obtained in the first evaluation of healthy controls,
G_HF_ the set of measure vectors obtained in the second evaluation of healthy controls,
G_WI_ the set of measure vectors obtained in the first evaluation of the waitlist control group,
G_WF_ the set of measure vectors obtained in the second evaluation of the waitlist control group.

The time intervals between the initial and final assessments are the same for all groups. The between-group Mahalanobis distances and the corresponding *P*_SAME_ (*G*_A_, *G*_B_) can be calculated using the procedures in Section “Calculation of *P*_SAME_ (*G*_A_, *G*_B_)” in Appendix. A treatment study is investigated by examining between-group Mahalanobis distances and their corresponding probabilities. The analysis begins by addressing the nine essential questions in Table 2.

**Table 2:** **Questions addressed in analysis of treatment effectiveness**.

1. Is there an adequate pre-treatment separation between the clinical population and the healthy controls?
2. Is the waitlist control group appropriately constructed?
3. Is the waitlist control group stable during the duration of the trial?
4. If there is a change in the waitlist control group, is it the result of continuing deterioration?
5. If there is a change in the waitlist control group, is it the result of spontaneous recovery?
6. Does the treatment group change during the trial?
7. If there is a change in the treatment group, is it due to continuing deterioration?
8. If there is a change in the treatment group is it due to spontaneous recovery?
9. Is there a positive response to treatment?

### Is there an adequate pre-treatment separation between the clinical population and the healthy controls?

The first step in the investigation is a model selection process that can identify the set of variables that discriminates between healthy and clinical populations. This is a critical step. As shown in section “Adding More Variables to a Diagnostic Discrimination Can, in Some Instances, Actually Reduce Classification Accuracy” and in Myers (87), Hastie et al. (5) and Zhao and Yu (114), inappropriate model selection can result in the unnecessary failure to construct an effective classifier. Several procedures can be considered. The essential requirement is to remove highly correlated variables that can degrade between-group discrimination. In this presentation D(G_X_, G_Y_) denotes a Mahalanobis distance. Mahalanobis distances are calculated using the procedure outlined in “Three Classifiers for Continuous Measures” in Appendix. If there is not a significant pre-treatment separation between these groups, as quantified by D(G_HI_, G_TI_), and a correspondingly small value of *P*_SAME_ (G_HI_, G_TI_), then the model selection process must be reviewed and the possibility of introducing other measures must be considered.

### Is the waitlist control group appropriately constructed?

D(G_WI_, G_TI_) is the Mahalanobis distance between the waitlist control group and the treatment group at initial assessment. The waitlist group and the treatment group meet the same inclusion/exclusion criteria. They should be indistinguishable. D(G_WI_, G_TI_) should be small or approximately zero. This gives criterion for testing the acceptability of the waitlist control group.

### Is the waitlist control group stable during the duration of the trial?

Mahalanobis distance D(G_WI_, G_WF_) quantifies change in the waitlist control group. In the absence of spontaneous recovery or continued deterioration, D(G_WI_, G_WF_) should be small. This provides a mechanism for investigating change in the absence of treatment, but an examination of D(G_WI_, G_WF_) alone does not identify possible changes in measures due to practice effects in neuropsychological tests or changes in physiological variables that result from increased comfortableness with EEG, MEG, or fMRI recording procedures in the second evaluation. Changes in the waitlist control group are assessed by addressing the next two questions.

### If there is a change in the waitlist control group, is it the result of continuing deterioration?

If the participants in the waitlist control group present continuing deterioration during the trial period, then D(G_WI_, G_WF_) can be large. Additionally, the separation between the waitlist control group and the healthy control group will increase giving D(G_WF_, G_HF_) > D(G_WI_, G_HI_).

### If there is a change in the waitlist control group, is it the result of spontaneous recovery?

If the waitlist control group presents recovery in the absence of treatment, then D(G_WI_, G_WF_) will be large, but in contrast with the preceding case, the separation between the waitlist control group and the healthy control group will decrease giving D(G_WI_, G_HI_) > D(G_WF_, G_HF_).

### Does the treatment group change during the trial?

D(G_TI_, G_TF_) is the pre-treatment versus post-treatment Mahalanobis distance. This is the multidimensional generalization of effect size (see “Calculation of Single-Variable Effect Size” in Appendix). In a successful treatment D(G_TI_, G_TF_) should be large and thus *P*_SAME_ (G_TI_, G_TF_) will be small. A large value of D(G_TI_, G_TF_) does not, however, establish a successful treatment. D(G_TI_, G_TF_) could have increased because of continued deterioration or spontaneous recovery. This motivates the next two questions.

### If there is a change in the treatment group, is it due to continuing deterioration?

If a large value of D(G_TI_, G_TF_) is due to continuing deterioration, we would expect the separation between the treatment group and the healthy control group to increase giving D(G_TF_, G_HF_) > D(G_TI_, G_HI_).

### If there is a change in the treatment group, is it due to spontaneous recovery?

If recovery has occurred, then D(G_TI_, G_TF_) is large and the separation between the treatment group and the control group will decrease giving D(G_TF_, G_HF_) < D(G_TI_, G_HI_). In the case of spontaneous recovery, as outlined above, a similar outcome would have been seen in the waitlist control group. In the limiting case of the complete absence of a treatment effect, the treatment group, and the waitlist control group should be statistically indistinguishable at the end of the trial giving a small value of D(G_TF_, G_WF_). This emphasizes the importance of a waitlist control group.

### Is there a positive response to treatment?

The post-treatment separation between the treatment group and the waitlist control group at the second assessment is given by D(G_TF_, G_WF_). It should increase in response to effective treatment in the absence of spontaneous recovery. To the degree that any one measure can assess treatment outcome it is D(G_TF_, G_WF_) and its corresponding *P*_SAME_(G_TF_, G_WF_). This is because this measure incorporates both the response to treatment and the effects of trial duration in the absence of treatment.

We suggest that answering these nine basic questions is the essential first step in the analysis process, but we also recognize that this is only the first step. If the answers to these questions suggest a positive response to treatment, then a detailed analysis of sources of variance is warranted.

### Longitudinal monitoring of an individual’s response to treatment

The calculations outlined thus far quantify between-group treatment responses. They are essential when validating the effectiveness of treatment. They do not, however, provide guidance concerning the progress of an individual patient. Let ${\underline{x}}_{Patient}$ be the vector of measures obtained from a patient. As argued in Section “A High Sensitivity and Specificity in a TBI Versus Control Population Classification does not Ensure Diagnostic Success when the Method is Applied in a More General Neuropsychiatric Population,” the non-specificity of many clinical measures, particularly psychophysiological variables, will probably prevent a diagnostic classification between different clinical groups. The longitudinal calculation of the probability that the patient is a member of the healthy control group, $P\left({\underline{\text{x}}}_{\mathrm{Patient}}|G_{\mathrm{Healthy}}\right)$, using methods described in “Three Classifiers for Continuous Measures” in Appendix might, however, provide a useful clinical measure. $P\left({\underline{\text{x}}}_{\mathrm{Patient}}|G_{\mathrm{Healthy}}\right)$ should increase during the course of a successful treatment. Optimism in this regard must be tempered by recalling the high session-to-session variability in CNS measures seen in some clinical populations. When a calculation is based on measures obtained from a single individual rather than on aggregate measures obtained from a population, this variability may make it impossible to use $P\left({\underline{\text{x}}}_{\mathrm{Patient}}|G_{\mathrm{Healthy}}\right)$ as a longitudinal measure. Further experience is required to evaluate the utility of $P\left({\underline{\text{x}}}_{\mathrm{Patient}}|G_{\mathrm{Healthy}}\right)$ in clinical practice.

Treatment response is often expressed in terms of effect size, and it is therefore helpful to show how effect sizes relate to the probability measures presented here. Effect sizes are defined in Section “Calculation of Single-Variable Effect Size” in Appendix. These definitions should be compared to the definition of Mahalanobis distance (see “Calculation of *P*_SAME_ (*G*_A_, *G*_B_)” in Appendix). It is seen that the between-group Mahalanobis distance for the special case of a single outcome measure (*Z* = 1) is the same as the Hedge’s *g* definition of effect size. It is also the same as the Cohen’s *d* definition when the number of members in each group is the same (*N*_A_ = *N*_B_). This identifies the first limitation of effect size as a measure of treatment. By definition, effect sizes consider only the *Z* = 1 case. Effect size cannot provide an assessment when several variables are used.

**Figure 4:**
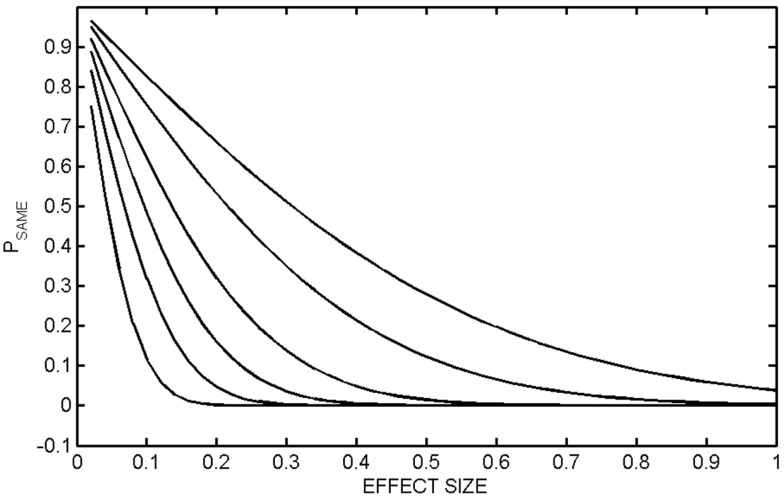
***P*_SAME_ (*G*_A_, *G*_B_) as a function of effect size**. *P*_SAME_ (*G*_A_, *G*_B_) was calculated as a function of effect size (equivalently the one-dimensional Mahalanobis distance) for different group sizes. In all calculations, the number of members in each group was the same, *N*_A_ = *N*_B_. The populations are *N*_A_ = *N*_B_ = 10 (top curve), 20, 50, 100, 200, 500 (bottom curve).

There is a further limitation of effect size that is not commonly recognized. Consider the equations for *P*_SAME_ (*G*_A_, *G*_B_), which we informally interpret in the context of treatment as the probability that Group A (pre-treatment) and Group B (post-treatment) are the same. These equations contain an explicit dependence on the number of members in each group, *N*_A_ and *N*_B_. Depending on *N*_A_ and *N*_B_, the same value of effect size, equivalently the same value of Mahalanobis distance, can give very different values of *P*_SAME_ (*G*_A_, *G*_B_). Examples are shown in Figure 4 where *P*_SAME_ (*G*_A_, *G*_B_) was calculated as a function of effect size for different population sizes. In these calculations *N*_A_ = *N*_B_. It is seen that the same value of effect size can result in very different between-group separations. Consider the case where effect size is 0.6. If *N*_A_ = *N*_B_ = 20, then *P*_SAME_ = 0.065. If *N*_A_ = *N*_B_ = 30, then *P*_SAME_ = 0.020 and *P*_SAME_ = 0.003 if *N*_A_ = *N*_B_ = 50. An effect size of 0.6 gives a strong indication of a positive effect, but only if there are 30 participants in each group, where we stress that this requires a total of 60 participants the study. Caution must be exercised even with an effect size of 0.6 if there are fewer than 40 participants in the study.

## A Procedure for Discriminating between Treatment Responders and Non-Responders Requires, Minimally, a Two Phase Investigation. This Procedure Must Include a Mechanism to Discriminate between Treatment Responders, Placebo Responders and Spontaneous Recovery

The analysis outlined in the previous section may be unacceptably simplistic. The treatment group may have two distinct outcome populations, a group that responds positively to treatment and a possibly larger group of non-responders. Similarly, the waitlist control group may have subpopulations that spontaneously recover, continue to deteriorate or are stable. The possibility of distinct subpopulations in the treatment group is particularly important. When all participants in the treatment group are included in the analysis, D(G_TF_, G_WF_) may be small, but this may obscure a very real positive clinical response in a subpopulation. While we cannot select through outcomes after the fact to get the results that we want, it is also important to avoid losing a treatment that could be significantly helpful to an appropriately selected population. Achieving this in a statistically responsible way requires a two phase investigation.

The Phase One investigation proceeds as outlined in the previous section. The criteria which will be used to distinguish between responders and non-responders should be established before this investigation is initiated. The seemingly simple process of identifying responders versus non-responders can be complex. How many measures should be used to make this determination? Incorporation of multiple outcome measures in a clinical trial can have significant and sometimes unanticipated consequences (88). Our emphasis here is not meeting statistical criteria required for regulatory clearance as was considered in Offen et al. but rather the identification of vectors of neuropsychological and psychophysiological variables that can separate populations. Let G_RI_ be the pre-treatment measure vectors obtained prior to treatment from the patients who proved to be treatment responders. Let G_NRI_ be the pre-treatment measure vectors that were from the non-responders. If Mahalanobis distance D(G_RI_, G_NRI_) is large, then the coefficient of determination (see “Coefficient of Determination” in Appendix) can be used to identify pre-treatment variables that separate responders and non-responders. These variables can then be used to construct a Phase Two investigation with patients who did not participate in the Phase One study and who meet responder criteria. If the first phase did indeed identify characteristics of responders, then the second study should have a high treatment response.

It is possible that the Phase One study simply identified individuals who were likely to experience a spontaneous recovery. This possibility can be investigated by comparing the characteristics of Phase One treatment responders with those members of the Phase One waitlist control group who recovered. If the measures that identify treatment responders are the same as the measures that characterize waitlist responders, then an argument can be made for a spontaneous recovery effect.

Further insights can be gained by including a placebo treatment group in the Phase One study, because this can clarify the distinction between a drug response and a drug effect (89). A drug response is a change that occurs after administering the drug. The drug effect is the portion of the response due to pharmacological action. It is the difference between the drug response and the placebo response. Similarly there is a distinction between a placebo response (the change that occurs after administration of the placebo) and the placebo effect which corrects for spontaneous recovery and regression to the mean (90). Determination of placebo effect therefore requires both a placebo treated group and a waitlist control group. If the identifying variables of active treatment responders and the identifying variables of waitlist and placebo responders are the same, then an argument can again be made for placebo recovery. It would, however, be a matter of interest, particularly in tests of psychotropics, to determine if the pre-treatment characteristics of placebo responders are different from the characteristics of responders in the active treatment and waitlist groups.

In summary, this two phase protocol can be used in an effort to identify subgroups in patient populations: (a) treatment responders versus non-responders, (b) patients who will recover spontaneously versus those who will not recover spontaneously, (c) patients who will deteriorate in the absence of treatment versus patients who will be clinically stable in the absence of treatment, and (d) placebo responders versus placebo non-responders.

## A Search for Prodromes of Delayed Onset Neuropsychiatric Disorders Following Traumatic Brain Injury Can be Implemented with These Procedures

A report published by the National Research Council and Institute of Medicine (91) defines a prodrome as “an early non-specific set of symptoms that indicates the onset of a disease before specific diagnosable symptoms occur.” A prodrome is not a risk factor. It is a manifestation of the disease itself. Costello and Angold (92) elaborate on this definition and noted that a prodromes may be non-specific “fever, malaise, headache and anorexia can be non-specific prodromes of infectious illness.” They continue “In summary, a prodrome is a premonitory manifestation of the disease. It is not a characteristic of the individual or their environment or a causal agent of the disease. A prodromal symptom may or may not continue to be manifest once the full disease appears. Conversely, the same disease may or may not manifest prodromal symptoms in different episodes.”

The search for prodromes of psychiatric illnesses has become a matter of intense interest. This activity follows from the recognition of the developmental nature of psychopathology. “… Second, psychiatry is beginning, at last, to take seriously the developmental nature of psychopathology. A recent national epidemiological study of adults in the United States reported that ‘Half of all lifetime cases start by age 14 and three fourths by age 24 years’ (93). This is a misestimate, caused by relying on retrospective recall by adults of their lifetime history of mental illness. Prospective studies beginning in childhood set the onset of most psychiatric disorders (apart from the dementias) in the first two decades of life (94, 95).” A neurodevelopmental etiology is important in this context because it suggests that prodromes may exist (96).

From a statistical point of view, the post-TBI population is a particularly promising population in a search for psychiatric prodromes because there is a high incidence of post-injury onset of psychiatric disorders. Rapp et al. (97) have reviewed the literature and found significantly increased incidences of depression, PTSD, generalized anxiety disorder, sleep disorders, and psychosis following traumatic brain injury. It is to be recognized, however, that the prodromes of, for example, depression following TBI may not be applicable in the general population since different pathophysiological mechanisms may be responsible. Nonetheless, it is an important population in its own right and given high incidence rates a good place to begin the search for prodromes of psychiatric disorders.

The statistical procedures outlined in previous sections can be used to search for prodromes of post-injury psychiatric illness. The process begins by collecting a set of measure vectors from TBI patients in the immediate post-injury period. These individuals are then followed longitudinally and two sets of participants, those positive for the disorder and those who do not present the disorder, are identified. *P*_SAME_ (see “Calculation of *P*_SAME_ (*G*_A_, *G*_B_)” in Appendix) is then calculated. If there is a statistically significant separation between these two sets, a systematic search for an optimal set of predictor variables can be performed with the coefficient of determination (see “Coefficient of Determination” in Appendix). It should be noted that the non-specificity of psychophysiological measures that are known to be altered in psychiatric illnesses may preclude finding prodromes for specific disorders. It may be that the best that can be achieved is an identification of individuals likely to present clinically in the absence of a prediction of the specific presentation, but this would still be of significant value.

## Discussion

Broadly stated, the four goals of laboratory medicine are diagnosis, longitudinal monitoring of treatment response or disease progression, detection of prodromes, and postmortem identification of the cause of demise. All of these objectives are, at core, classification problems. This contribution has considered the first three objectives with a focus on traumatic brain injury. As observed earlier, traumatic brain injury presents particularly demanding assessment challenges. Six conclusions have been developed in this paper.

It was shown that a statistically significant separation of a clinical population and an appropriately matched healthy comparison group does not ensure a successful diagnostic procedure. It is necessary but not sufficient. While this is well established in the technical literature, it is not always recognized in the clinical community.

The post-TBI population is clinically heterogeneous. Different injury events can initiate different pathophysiological processes. It therefore follows that there will never be a single test for traumatic brain injury. A multidimensional assessment is required. The incorporation of additional measures into a multivariate discrimination must, however, be undertaken with care. Contrary to common understanding, adding variables to a classifier can in some instances degrade performance. We provided an example of an EEG classifier where the error rate decreased from 65% (actually worse that chance) to 27% as measures were eliminated from the discrimination.

Reports of diagnostic sensitivity and specificity must also be considered with care. Assessment procedures, for example neuropsychological evaluations and psychophysiological measures such as heart rate variability and event related potentials, may be sensitive to CNS pathology, but the emerging literature indicates that they are non-specific. While a set of measures may be able to distinguish between healthy controls and TBI patients in a carefully constructed clinical trial, these measures alone may well not be able to distinguish between traumatic brain injury, bipolar disorder or major depressive disorder. Since the clinical response will be very different, this lack of specificity is not merely an academic consideration. That said, this does not constitute an argument against neuropsychological and psychophysiological assessments in neuropsychiatry. Measurement of body temperature provides a valuable example. Body temperature is a non-specific clinical measure but it is nonetheless a central element in any clinical evaluation. Measures of CNS coherence, synchronization, causal pathways, and network geometry are known to be non-specific but will, we suggest, become increasingly important in neuropsychiatric practice.

A study of treatment effectiveness must be responsive to the high degree of intra-individual longitudinal variability of biological measures obtained in neuropsychiatric populations. This, we have argued, is particularly true of TBI patients. Systematic test-retest reliability studies are essential. Additionally, the high incidence of spontaneous recovery from neuropsychiatric disorders, including TBI, establishes the importance of waitlist control groups. While a waitlist control group is methodologically valuable, it is also recognized that utilizing a waitlist group can raise important ethical questions (98, 99). The waitlist can be detrimental. Depending on the clinical presentation and the duration of the delay, significant deterioration can occur. Devilly and McFarlane (100) suggested performing comparisons with existing waitlist control data, but this possibility is limited to studies that have common inclusion/exclusion criteria and common outcome measures. As in all research involving human participants, ideal statistical design must be subordinated to considerations of responsible clinical behavior.

The heterogeneity of these clinical populations also suggests that for any given treatment there may be responder and non-responder subgroups in the intake populations. The responder subgroup may be small and a treatment that might be highly effective for that group may be lost in aggregate statistics. But we cannot post-facto sort through outcomes looking for the results that we want to see and declare a success. Positive response could be the result of a placebo effect or spontaneous recovery. At a minimum, a two phase study is required where the characteristics of responders are identified in the first phase. These characteristics are then used as inclusion/exclusion criteria for a second study which should show a high positive response rate. This second study should include a placebo treatment arm.

A virtue of a statistical analysis of treatment response is the potential for characterizing partial recovery. $P\left({\underline{\text{x}}}_{\mathrm{Patient}}|G_{\mathrm{Healthy}}\right)$ can be computed in the absence of a diagnosis and provides a global assessment of an individual’s response to treatment. If a diagnosis has been made, the probability of membership in the patient’s diagnosis group will hopefully decrease but typically it will remain non-zero. Assessments made in reference to a single diagnostic group must be understood with care because symptoms can be present in the definition of more than one diagnostic group. For example, symptoms present in post concussion syndrome are also found in PTSD, depression and, most pertinently, in healthy control populations. A review of the endorsement frequency of post concussion symptoms in populations that do not have a history of TBI found that in some studies endorsement rates in healthy controls were equal to or greater than endorsement frequencies in populations with a history of mild TBI (101).

A further complication must be recognized. Neuropsychiatric disorders are not single factor entities. The Potter et al. (102) study of post concussion symptoms found three subscales. Similarly, the Beck Depression Index identifies three subscales (103, 104), and the Pittsburgh Sleep Quality Index has seven subscales (105). Statistical results indicating partial recovery may reflect a very positive response on some subscales and not on others. A naïve statistical analysis that does not recognize this possibility will not capture these clinically important findings.

A great deal of attention is now being directed to the search for prodromes of neuropsychiatric disorders. We have shown that this can be constructed as a classification problem that utilizes the results of a longitudinal study.

It has been suggested that imaging studies, genomic investigations, plasma biomarker data, neuropsychological evaluations, and psychophysiological measures can be combined to construct quantitatively informed treatments specific to the individual patient. The utility of these measures in making between-group discriminations, for example, evaluating treatment effect size, is established. Our assessment of the utility of these measures for guiding individual treatment is more guarded. The heterogeneity of the populations, low specificity and low test-retest reliability of these measures argues against unrestrained optimism about their value at the individual rather than group level. When essential and often neglected statistical safeguards are introduced, previously reported positive results are found to be unsubstantiated. While progress in the longitudinal quantitative assessment of individual patients can certainly be made, statistical caution must be exercised.

## Conflict of Interest Statement

The authors declare that the research was conducted in the absence of any commercial or financial relationships that could be construed as a potential conflict of interest.

## Appendix

### A. Calculation of *P*_SAME_ (*G*_A_, *G*_B_)

Applied Multivariate Statistics [Johnson and Wichern (106), p. 210]. Panel on Discriminant Analysis, Classification and Clustering [(107), p. 38].

#### Assumptions used in the derivation of *P*_SAME_ (*G*_A_, *G*_B_)

- The two populations given the class label, both Group A and Group B, are multivariate normal,
- the population covariance matrices are the same,
- *N*_A_ + *N*_B_ > *Z* + 1, where *Z* is the number of variables in the discrimination (the dimension of the measure vector).

For Group A let ${\underline{\hat{\mu }}}_{\text{A}}=({\hat{\mu }}_{\text{A}1},\;{\hat{\mu }}_{\text{A}2},\;\ldots \;{\hat{\mu }}_{\text{AZ}})$ denote the vector of sample mean values.

$${\hat{\mu }}_{\mathrm{Ai}}=\frac{1}{N_{A}}\sum_{m=1}^{N_{\text{A}}}\;X_{i}\left(m\right)$$

where *x*_i_(*m*) is the *m*-th value of discriminating variable *i* in Group A. ${\left({\hat{\sigma }}_{A}^{2}\right)}_{\mathrm{i,}\;j}$ is element (*i*, *j*) of the Group A sample covariance matrix.

$${\left({\hat{\sigma }}_{A}^{2}\right)}_{\mathrm{i,j}}=\frac{1}{N_{A}-1}\overset{N_{A}}{\underset{m=1}{\;\sum }}\left(x_{i}\left(m\right)-{\hat{\mu }}_{\mathrm{Ai}}\right)\left(x_{j}\left(m\right)-{\hat{\mu }}_{\mathrm{Aj}}\right)$$

∑_A_ denotes the *Z* × *Z* matrix of elements ${\left({\hat{\sigma }}_{A}^{2}\right)}_{i,j}$, and $\sum_{A}^{-1}$ denotes its inverse. These quantities are defined analogously for Group B. ${\left({\hat{\sigma }}_{\mathrm{A,B}}^{2}\right)}_{i,j}$ is element (*i*, *j*) of the between-group sample covariance matrix.

$${\left({\hat{\sigma }}_{\mathrm{A,B}}^{2}\right)}_{\mathrm{i,j}}=\frac{\left(N_{A}-1\right){\left({\hat{\sigma }}_{A}^{2}\right)}_{\mathrm{i,j}}+\left(N_{B}-1\right){\left({\hat{\sigma }}_{B}^{2}\right)}_{\mathrm{i,j}}}{N_{A}+N_{B}-2}$$

∑_A,B_ denotes the matrix formed by these elements, and $\sum_{\mathrm{A,B}}^{-1}$ denotes its inverse. The between-group Mahalanobis distance is given by

$$D_{\mathrm{A,B}}^{2}={\left(\begin{array}{c} {\hat{\mu }}_{\mathrm{A1}}-{\hat{\mu }}_{\mathrm{B1}}\\ {\hat{\mu }}_{\mathrm{A2}}-{\hat{\mu }}_{\mathrm{B2}}\\ \vdots \\ {\hat{\mu }}_{\mathrm{AZ}}-{\hat{\mu }}_{\mathrm{BZ}} \end{array}\right)}^{T}\sum_{\mathrm{A,B}}^{-1}\left(\begin{array}{c} {\hat{\mu }}_{\mathrm{A1}}-{\hat{\mu }}_{\mathrm{B1}}\\ {\hat{\mu }}_{\mathrm{A2}}-{\hat{\mu }}_{\mathrm{B2}}\\ \vdots \\ {\hat{\mu }}_{\mathrm{AZ}}-{\hat{\mu }}_{\mathrm{BZ}} \end{array}\right)$$

For the special case where the discrimination is based on a single variable (*Z* = 1 in our notation), the expression for Mahalanobis distance is given by

$$D_{\mathrm{A,B}}^{2}=\frac{{\left({\hat{\mu }}_{A}-{\hat{\mu }}_{B}\right)}^{2}}{{\hat{\sigma }}_{\mathrm{A,B}}^{2}}$$

$${\hat{\sigma }}_{\mathrm{A,B}}^{2}=\frac{\left(N_{A}-1\right){\hat{\sigma }}_{A}^{2}+\left(N_{B}-1\right){\hat{\sigma }}_{B}^{2}}{N_{A}+N_{B}-2}$$

${\hat{\mu }}_{A}$ is the Group A sample mean for this single variable and ${\hat{\sigma }}_{A}$ is the sample standard deviation of that mean. ${\hat{\mu }}_{B}$ and ${\hat{\sigma }}_{B}$ are defined analogously.

*P*_SAME_ (*G*_A_, *G*_B_) is given by an *F*-test

$$P_{\mathrm{SAME}}\left(G_{A},G_{B}\right)=I_{\frac{\nu_{2}}{\nu_{2}+\nu_{1}F}}\left(\frac{\nu_{2}}{2},\frac{\nu_{1}}{2}\right)$$

ν_1_ = *Z*, the number of discriminating variables, and ν_2_ = *N*_A_ + *N*_B_ − *Z* − 1.

$$F=\frac{N_{A}N_{B}\left(N_{A}+N_{B}-Z-1\right)D_{\mathrm{A,B}}^{2}}{\left(N_{A}+N_{B}\right)\left(N_{A}+N_{B}-2\right)Z}$$

*I*_X_(a, b) is the incomplete β function.

$$I_{x}\left(a,b\right)=\frac{1}{B\left(a,b\right)}\overset{x}{\underset{0}{\int }}t^{a-1}{\left(1-t\right)}^{b-1}dt$$

and B(a, b) is the β function.

$$B_{\left(\mathrm{a,b}\right)}=\overset{1}{\underset{0}{\int }}t^{\mathrm{a-1}}{\left(1-t\right)}^{\mathrm{b-1}}dt$$

*P*_SAME_ is seen to be monotone decreasing with Mahalanobis distance. From the numerator of *F* it is seen that *N*_A_ + *N*_B_ > *Z* + 1 is a requirement of the analysis.

#### Interpretation of *P*_SAME_

*P*_SAME_ is only meaningful in the context of a two group comparison. *P*_SAME_ is the probability under the null hypothesis $\left({\underline{\mu }}_{\text{A}}={\underline{\mu }}_{\text{B}}\right)$ that an observed Mahalanobis distance will be at least as large as the value of $D_{\mathrm{A,B}}^{2}$ used to calculate *P*_SAME_. Operationally, a large value of the Mahalanobis distance results in a small value of *P*_SAME_ which is evidence against the null hypothesis and which therefore suggests that $\left({\underline{\mu }}_{\text{A}}\neq {\underline{\mu }}_{\text{B}}\right)$. As a general observation, *p*-values should only be used as evidence against a null hypothesis. A presentation of the statistically valid understanding of *p*-values as random variables is given in Murdoch et al. (6).

Elements of ∑_A_ and ∑_B_ the Group A and Group B covariance matrices, are used in the calculation of the between-group covariance matrix ∑_A,B_ which is then used to calculate $D_{\mathrm{A,B}}^{2}$. Since potentially different group-specific covariance matrices are used to calculate $D_{\mathrm{A,B}}^{2}$, the rationale for an assumption of equal group covariance matrices cited above may be unclear. This assumption follows from the use of a Wishart distribution, which requires equal covariances, to derive the expression for *P*_SAME_. That is, the assumption of equal covariances is not necessary to compute $D_{\mathrm{A,B}}^{2}$ but to derive the expression for the probability. The requirement of equal covariances is not typically observed in practice. Therefore, though it is not usually emphasized, *P*_SAME_ is a best available approximation.

### B. Calculation of *P*_ERROR − FORMULA_ (*G*_A_, *G*_B_)

Johnson and Wichern [(106), p. 598] Applied Multivariate Statistics. Wasserman (8) All of Statistics (Theorem 22.5).

#### Assumptions used in the derivation of *P*_ERROR − FORMULA_ (*G*_A_, *G*_B_)

- The two populations given the class label are multivariate normal.
- The population covariance matrices are the same.
- The means and covariances are known. In the present context, “known” indicates that the numerical estimates of means and covariances used in the calculations are assumed to be exact.
- The prior probability of observing either class 1/2.

#### Interpretation of *P*_ERROR − FORMULA_ (*G*_A_, *G*_B_)

*P*_ERROR_ is only meaningful in a two group classification. It is an estimate of the error rate obtained in a dichotomous Group A versus Group B classification. This is the optimal Bayes classifier and is the best available prediction of classification error if only means and covariances are known. As discussed in the text, it can be a serious underestimate of the true error rate.

Using the previously stated expression for $D_{\mathrm{A,B}}^{2}$, *P*_ERROR − FORMULA_ (*G*_A_, *G*_B_) is given by:

$$P_{\mathrm{ERROR-FORMULA}}\left(G_{A},G_{B}\right)=1-\Phi \left(\frac{\sqrt{D_{\mathrm{A,B}}^{2}}}{2}\right)=\Phi \left(\frac{-\sqrt{D_{\mathrm{A,B}}^{2}}}{2}\right)$$

Φ(*x*) is the cumulative normal distribution

$$\Phi \left(x\right)=\frac{1}{\sqrt{2\pi }}\overset{x}{\underset{-\infty }{\int }}e^{-u^{2}∕2}du=\frac{1}{2}\left[1+\mathrm{erf}\;\left(\frac{x}{\sqrt{2}}\right)\right]$$

Where erf(*x*) is the error function

$$\mathrm{erf}\left(x\right)=\frac{2}{\sqrt{\pi }}\overset{x}{\underset{0}{\int }}e^{-t^{2}}dt$$

For the case of unequal priors, the expression for *P*_ERROR − FORMULA_ (*G*_A_, *G*_B_) becomes

$$\begin{array}{llll} & P_{\mathrm{ERROR-FORMULA}}=p_{A}\Phi \left(-\frac{1}{2}\sqrt{D_{\mathrm{A,B}}^{2}}+\frac{1}{\sqrt{D_{\mathrm{A,B}}^{2}}}\log_{e}\left(\frac{p_{B}}{p_{A}}\right)\right)\; & & \;\\ & \;+p_{B}\Phi \left(-\frac{1}{2}\sqrt{D_{\mathrm{A,B}}^{2}}-\frac{1}{\sqrt{D_{\mathrm{A,B}}^{2}}}\log_{e}\left(\frac{p_{B}}{p_{A}}\right)\right)\; & & \; \end{array}$$

where *p*_A_ and *p*_B_ are prior probabilities. We emphasize that in the derivation the logarithm was used to remove exponents in the density function and is the natural logarithm.

### C. Three classifiers for continuous measures

#### Classification using minimum Mahalanobis distance and $P_{\mathrm{ABS}}\left({\underline{\text{x}}}_{\mathrm{TEST}},\;G_{A}\right)$

For classification problems involving an arbitrary number of groups, it is possible to classify measure vectors based on the minimum Mahalanobis distance. Let ${\underline{x}}_{\mathrm{TEST}}$ be the vector of *Z* measures obtained from a single individual. In a clinical study this could be the set of results obtained from a patient.

$${\underline{x}}_{\mathrm{TEST}}=\left(x_{1\mathrm{-Test}},x_{2-\mathrm{Test}},\cdots \cdots x_{Z-\mathrm{Test}}\right)$$

The Mahalanobis distance between ${\underline{x}}_{\mathrm{TEST}}$ and Group A is given by

$$D_{\mathrm{Test,A}}^{2}={\left(\begin{array}{c} x_{1-\mathrm{Test}}-{\hat{\mu }}_{\mathrm{A1}}\\ x_{\mathrm{2-Test}}-{\hat{\mu }}_{\mathrm{A2}}\\ \vdots \\ x_{\mathrm{Z-Test}}-{\hat{\mu }}_{\mathrm{AZ}} \end{array}\right)}^{T}\sum_{A}^{-1}\left(\begin{array}{c} x_{1-\mathrm{Test}}-{\hat{\mu }}_{\mathrm{A1}}\\ x_{\mathrm{2-Test}}-{\hat{\mu }}_{\mathrm{A2}}\\ \vdots \\ x_{\mathrm{Z-Test}}-{\hat{\mu }}_{\mathrm{AZ}} \end{array}\right)$$

${\hat{\mu }}_{\mathrm{Ai}}$ is the mean value of the *i*-th measure calculated from the members of Group A. ∑_A_ is the previously specified Group A covariance matrix, and $\sum_{A}^{-1}$ is its inverse. Measure vector ${\underline{x}}_{\mathrm{TEST}}$ is classified into Group J if

$$D_{\mathrm{Test,}\;J}^{2}=\min\left\{D_{\text{Test},\text{ I}}^{2},\;I=1,2,\;\ldots ,\;K\right\}$$

This provides a classification but does not give an estimate of the probability that ${\underline{x}}_{\mathrm{TEST}}$ is a member of Group J. This is provided by *P*_ABS_. The assumptions underlying the calculation of *P*_ABS_ are the same as those underlying the calculation of *P*_SAME_. In the case of *P*_ABS_ one of the two populations has a single member [(10), p. 136]. Therefore, it is necessary to assume that the population of Group A is multivariate normal, but since this is a hypothesis test, it is not necessary to assume that means and covariances are known exactly.

#### Assumption in the derivation of $P_{\text{ABS}}({\underline{\text{x}}}_{\text{Test}}|G_{\text{A}})$

The population given the class label is multivariate normal. Because $P_{\text{ABS}}({\underline{\text{x}}}_{\text{Test}}|G_{\text{A}})$ is calculated separately for each group, *I* = 1, 2, … *K*, it is not necessary to assume the group covariances are equal.

#### Interpretation of $P_{\mathrm{ABS}}\left({\underline{x}}_{\mathrm{Test}}|G_{I}\right)$

*P*_ABS_ must be interpreted with care. This is the *a priori* marginal probability. A small value is evidence that the individual is not likely to be a member of that group but a large value should not be used as evidence that an individual is from that group. Given the dependence of *P*_ABS_ on Mahalanobis distance, this is the same assignment as that obtained with the minimum Mahalanobis distance, but we now have a sense of how likely that membership is.

$$P_{\mathrm{ABS}}\left({\underline{x}}_{\mathrm{TEST}}|G_{A}\right)=I_{\frac{\nu_{2}}{\nu_{2}+\nu_{1}F}}\left(\frac{\nu_{2}}{2},\frac{\nu_{1}}{2}\right)$$

where *v*_1_ = *Z*, the number of discriminating variables, and *v*_2_ = *N*_A_ − *Z* where *N*_A_ is the number of members in Group A. For the case when *N*_B_ = 1, *F* is given by

$$F=\frac{N_{A}\left(N_{A}-Z\right)}{\left(N_{A}^{2}-1\right)Z}D_{\mathrm{TEST,A}}^{2}$$

It is seen that this is equivalent to *P*_SAME_ (*G*_A_, *G*_B_) for the special case *N*_B_ = 1 and ${\hat{\underline{\mu }}}_{\text{B}}={\underline{x}}_{\mathrm{TEST}}$. Given the dependence of *P*_ABS_ on Mahalanobis distance, classification by maximum *P*_ABS_ is equivalent to classification by minimum Mahalanobis distance.

#### Classification using Bayesian maximum likelihood $P_{\text{BAYES}}({\underline{\text{x}}}_{\text{TEST}},G_{\text{A}})$

McLachlan (108) p. 53.

The Bayes classifier can accommodate unequal prior probabilities. Let $p_{\text{M}}^{\prime }$ denote the prior probability of membership in Group M.

#### Assumptions in the derivation of *P*_BAYES_

The populations given class labels are multivariate normal. It is not necessary to assume that covariances are equal or that the prior probabilities are equal.

#### Interpretation of *P*_BAYES_

${\underline{x}}_{\mathrm{TEST}}$ is classified to the group that has the largest value of *P*_BAYES_.

The group-specific density estimate of ${\underline{x}}_{\mathrm{TEST}}$ in Group A is

fA(x¯TEST)=1(2π)Z∕2|ΣA|1∕2exp−12x1−TEST−μ^A1x2−TEST−μ^A2⋮xZ−TEST−μ^AZT∑A−1x1−TEST−μ^A1x2−TEST−μ^A2⋮xZ−TEST−μ^AZ

where ∑_A_ is the Group A covariance matrix, |⋅| indicates the determinant, and $\Sigma_{A}^{-1}$ is the inverse covariance matrix. Using Bayes’ theorem, the posterior probability of ${\underline{x}}_{\mathrm{TEST}}$ is

$$P_{\mathrm{BAYES}}\left({\underline{x}}_{\mathrm{TEST}}|G_{A}\right)=\frac{p_{A}^{\prime }f_{A}\left({\underline{x}}_{\mathrm{TEST}}\right)}{\overset{K}{\underset{M=1}{\sum }}p_{M}^{\prime }f_{M}\left({\underline{x}}_{\mathrm{TEST}}\right)}$$

*K* is the number of distinct groups in the classification problem, $p_{M}^{\prime }$ is the prior probability of membership in the Group M, and $p_{A}^{\prime }$ is prior probability of membership in Group A. ${\underline{x}}_{\mathrm{TEST}}$ is classified into the group giving the largest values of *P*_BAYES_.

#### Classification using quadratic classifiers

Classification by maximum Bayes likelihood is equivalent to classification by a quadratic classifier.

$$\begin{array}{llll} & \log_{e}\left\{{\left(2\pi \right)}^{Z∕2}p_{A}^{\prime }f_{A}\left({\underline{x}}_{\mathrm{TEST}}\right)\right\}=-\frac{1}{2}\log_{e}|\Sigma_{A}|\; & & \;\\ & \;-\frac{1}{2}{\left(\begin{array}{c} x_{1-\mathrm{TEST}}-{\hat{\mu }}_{\mathrm{A1}}\\ x_{2-\mathrm{TEST}}-{\hat{\mu }}_{\mathrm{A2}}\\ \vdots \\ x_{Z-\mathrm{TEST}}-{\hat{\mu }}_{\mathrm{AZ}} \end{array}\right)}^{T}\sum_{A}^{-1}\left(\begin{array}{c} x_{1-\mathrm{TEST}}-{\hat{\mu }}_{A1}\\ x_{2-\mathrm{TEST}}-{\hat{\mu }}_{\mathrm{A2}}\\ \vdots \\ x_{Z-\mathrm{TEST}}-{\hat{\mu }}_{\mathrm{AZ}} \end{array}\right)+\log_{e}p_{A}^{\prime }\; & & \; \end{array}$$

The right hand side expression is the quadratic discriminant function, denoted by$Q\left({\underline{x}}_{\mathrm{TEST}},A\right)$. ${\underline{x}}_{\mathrm{TEST}}$ is classified into the group giving the greatest value of that function. The assumptions of the quadratic classifier are therefore the same as the Bayes classifier. The populations are multivariate normal, but an assumption of equal covariances is not required.

### D. Simulation studies comparing *P*_ERROR − EMPIRICAL_ and *P*_ERROR − FORMULA_

As presented in Calculation of *P*_ERROR − FORMULA_ (*G*_A_, *G*_B_), the error rate in a two group classification can be estimated by a formula. We denote this estimate by *P*_ERROR − FORMULA_. The error rate can also be determined empirically. This is denoted by *P*_ERROR − EMPIRICAL_. There is more than one procedure for obtaining an empirical estimate of classification error. Two commonly used methods are the *k*-fold cross validation and the out-of-sample validation. In the case of an out-of-sample validation, the training sets are specified and the classifier is constructed once. Additional elements of known group membership that are not present in the training sets are then classified. The classifier is unchanged throughout the validation process. In the case of a *k*-fold cross validation, elements are withdrawn from the existing sample, the classifier is constructed in their absence and the withdrawn elements are classified using the classifier. The process is repeated. The withdrawn elements are restored to the classifier, a different set of *k* elements are withdrawn and these elements are classified with a reconstructed classifier. *k*-fold cross validation requires specification of the parameter *k*. In part this choice turns on the computational resources available. An *N*-fold classification, *k* = *N*, where each element of the classifier is removed and classified is the definitive implementation of a *k*-fold cross validation. In addition to accuracy, the *k*-fold cross has another virtue over formula determined error rates. *P*_ERROR − FORMULA_ (*G*_A_, *G*_B_) provides an error estimate for two group classifications. A *k*-fold cross validation can be used to assess classifications across an arbitrary number of groups provided that the removed measure vectors are randomly drawn from all groups in the classification.

Is *P*_ERROR − EMPIRICAL_ always greater than or approximately equal to *P*_ERROR − FORMULA_, where *P*_ERROR − EMPIRICAL_ is determined by a *k*-fold cross validation? This question was investigated by a series of simulation studies. When evaluating the results of these simulations a technical point concerning the validity of *P*_ERROR − FORMULA_ should be considered. These studies will be favorable to *P*_ERROR − FORMULA_ because the data were generated in conditions where *P*_ERROR − FORMULA_ holds; that is, the data really are near-Gaussian (108). Thus in the limit of more and more data, the sample means and covariances will converge on true means and covariances, and *P*_ERROR − FORMULA_ becomes an almost exact error rate. This produces agreement between the formula-predicted error rates and the empirically determined error rates for large data sets. In a real world application where the true distributions are not Gaussian, the hypotheses used in the derivation of *P*_ERROR − FORMULA_ do not hold and *P*_ERROR − FORMULA_ gives a worse estimate. These simulations therefore make *P*_ERROR − FORMULA_ look better than it really is. If *P*_ERROR − FORMULA_ is an underestimate in simulations, its accuracy in real world applications is probably even worse.

The first simulation results were obtained in a one-dimensional discrimination using the one-dimensional expressions for Mahalanobis distance and joint covariance shown in Calculation of *P*_SAME_ (*G*_A_, *G*_B_). Normally distributed data sets were generated computationally with means approximately equal to 3.5 and −3.5 and with standard deviations for both distributions approximately equal to 15. Population numbers *N*_A_ and *N*_B_ were the same in each simulation. The empirical error rate was determined in a *k*-fold cross validation. One thousand simulations were performed for each set of *N*_A_ and *N*_B_ values. The results are displayed in the table. *F*_E > F_ denotes the fraction of cases where the empirical estimate of error was greater than the estimate computed from the formula. The results are summarized in Table A1.

**Table A1:** **Classification error rate in a univariate simulation.**.

*N*_A_, *N*_B_	<*P*_SAME_ >	<*P*_ERROR − FORMULA_>	<*P*_ERROR − EMPIRICAL_>	*F*_E > F_
10	0.35659	0.37104	0.44250	0.712
20	0.25619	0.39337	0.42218	0.642
30	0.17765	0.39831	0.41356	0.607
40	0.13235	0.40107	0.41074	0.587
50	0.10093	0.40320	0.41194	0.569
100	0.02239	0.40685	0.40989	0.548
150	0.00501	0.40762	0.40957	0.533
200	0.00121	0.40793	0.40941	0.534
250	0.00023	0.40799	0.40927	0.537
300	0.00005	0.40815	0.40884	0.517
350	0.6 × 10^−5^	0.40801	0.40854	0.517
400	0.2 × 10^−5^	0.40835	0.40876	0.515
450	0.4 × 10^6^	0.40831	0.40853	0.513
500	0.1 × 10^−6^	0.40829	0.40860	0.507

N_A_ = *N*_B_ is the number of members in each group. <*P*_SAME_> is the average of *p*-values obtained in an *F*-test. <*P*_ERROR − FORMULA_> is the average predicted classification error rate given the assumption of normal distributions and equal covariances. <*P*_ERROR − EMPIRICAL_> is the classification error rate determined in a *k*-fold cross validation. *F*_E > F_ is the fraction of cases where the empirical estimate of error was greater than the estimate computed from the formula. Averages were obtained from 1,000 simulations for each *N*_A_ = *N*_B_ pair.

As would be expected the value of *P*_SAME_ averaged over 1,000 simulations is monotone decreasing with *N*_A_ and *N*_B_ and is large when the population numbers are small. In the case of this one-dimensional discrimination, the exhaustive *k* = *N* cross validation classification error rates and the formula estimated error rates are in substantial agreement for *N*_A_ = *N*_B_ ≥ 50. The average normalized difference between the two values ranges from 20.8% for *N*_A_ = *N*_B_ = 10 to 6.5% for *N*_A_ = *N*_B_ = 50, and to 1.8% for *N*_A_ = *N*_B_ = 500 (The normalized difference is the difference between the empirically determined error rate and the formula determined error rate divided by their average. The normalized difference is determined in each of the 1,000 simulations. The average normalized difference is reported here). It is seen that even in the case of small values of *N*_A_ and *N*_B_ the empirically determined error rate is approximately the same or larger than *P*_ERROR − FORMULA_.

A greater divergence between *P*_ERROR − FORMULA_ and *P*_ERROR − EMPIRICAL_ was seen in a two dimensional classification. In the two dimensional simulations, two procedures were used for calculating *P*_ERROR − FORMULA_. In one version, *P*_ERROR − FORMULA_ is calculated in the absence of exact knowledge of the underlying distributions. It is calculated using an approximation of the distributions’ mean values and covariance matrices calculated using the available sampled data. This is designated by *P*_ERROR − FORMULA − SAMPLE_ (This was not done in the previously reported one-dimensional simulations.). In the other version, *P*_ERROR − FORMULA_ is calculated using the exact specifications of the distributions from the parameters that were used by the algorithm to generate the experimental distributions. This is designated as *P*_ERROR − FORMULA − OPTIMAL_ and is only available in simulation studies. It can be shown that this error estimate is optimal in the sense that a classifier not built with the population values will perform less well [(8), p. 352, Theorem 22.5]. For practical purposes in studies using observed data, *P*_ERROR − FORMULA − OPTIMAL_ is not available, and judgments must be based on *P*_ERROR − FORMULA − SAMPLE_. In the two dimensional classification simulations report *P*_ERROR − EMPIRICAL_ > *P*_ERROR − FORMULA − SAMPLE_ in 94% of the cases and *P*_ERROR − EMPIRICAL_ > *P*_ERROR − FORMULA − OPTIMAL_ in 54% of the cases.

The simulation in *ℜ*^2^ and considers two bivariate normal distributions.

Distribution A: ${\underline{\mu }}_{\text{A}}=\left(0,0\right)$ and Cov_A_ = *I*_2 × 2_. ${\underline{\mu }}_{\text{A}}$ is the vector of means, Cov_A_ is the within-group covariance matrix, and is *I*_2 × 2_ the two dimensional identity matrix.

Distribution B: ${\underline{\mu }}_{\text{B}}=(0.01,0.01)$ and Cov_B_ = *I*_2 × 2_

#### Construct training set $\left\{\underline{X}\right\}=\left\{{\underline{x}}_{1},{\underline{x}}_{2},\cdots \cdots {\underline{x}}_{20}\right\}$

At random choose Distribution A or Distribution B. Draw randomly an element from the chosen distribution. This will be ${\underline{x}}_{1}={ℜ}^{2}$. Repeat this procedure 19 times; select either distribution in a random process and draw at random an element from the selected distribution. The resulting set $\left\{\underline{X}\right\}$, will have 20 elements with approximately 10 elements from Distribution A and Distribution B.

#### Build the classifier

Find the mean of all ${\underline{x}}_{j}\in \left\{\underline{X}\right\}$ such that ${\underline{x}}_{j}$ is an element of Distribution A. This vector of means is ${\hat{\underline{\mu }}}_{\text{A}}$ where the superscript indicates that the vector was determined by the data in $\left\{\underline{X}\right\}$. Calculate the covariance matrix $Ĉov_{A}$from those elements of $\left\{\underline{X}\right\}$ that were drawn from Distribution A. Similarly calculate ${\hat{\underline{\mu }}}_{\text{B}}$ and $Ĉov_{B}$. The between-group covariance matrix is calculated from $Ĉov_{A}$ and $Ĉov_{B}$ and the $\left\{\underline{X}\right\}$ population values *N*_A_ and *N*_B_.

#### Compute *P*_ERROR − FORMULA − SAMPLE_

The between-group Mahalanobis distance, ${\hat{D}}_{\mathrm{AB}}^{2}$, is calculated from ${\hat{\underline{\mu }}}_{\text{A}}$, $Ĉov_{A}$, ${\hat{\underline{\mu }}}_{\text{B}}$, $Ĉov_{B}$, *N*_A_, and *N*_B_. *P*_ERROR − FORMULA − SAMPLE_ is calculated using the optimal error rate for unequal priors.

#### Compute *P*_ERROR − FORMULA − OPTIMAL_

In this case, the between-group Mahalanobis distance is computed using the known exact values of ${\hat{\underline{\mu }}}_{\text{A}}$, ${\hat{\underline{\mu }}}_{\text{B}}$, Cov_A_, and Cov_B_, and the values of *N*_A_ and *N*_B_ established by the random draw that constructed $\left\{\underline{X}\right\}$. *P*_ERROR − FORMULA − OPTIMAL_ is calculated using this value of the Mahalanobis distance and the previous equation.

#### Calculate *P*_ERROR − EMPIRICAL_

This is the cross validation error. Start with the 20 element set $\left\{\underline{X}\right\}$. In a random assignment, place each element of $\left\{\underline{X}\right\}$ in set *S*_1_, *S*_2_, …. or *S*_5_. Each element of $\left\{\underline{X}\right\}$ is assigned to only one set *S*. Thus each set *S* contains four elements. Remove *S*_1_ from $\left\{\underline{X}\right\}$ to give $\left\{\underline{X}\right\}-S_{1}$, this set has 16 elements. Construct the classifier with $\left\{\underline{X}\right\}-S_{1}$ using the known identities of the elements in this set. Classify all elements of S_1_ and determine the number of errors, NE_1_, where 0 ≤ NE_1_ ≤ 4. This process is repeated for sets *S*_2_, …, *S*_5_ to determine NE_2_, …, NE_5_. Calculate *P*_ERROR − EMPIRICAL_.

$$P_{\mathrm{ERROR-EMPIRICAL}}=\frac{NE_{1}+NE_{2}+\cdots \cdots +NE_{5}}{20}$$

Using this procedure *P*_ERROR − EMPIRICAL_ was determined 1,000 times. In this simulation *P*_ERROR − EMPIRICAL_ > *P*_ERROR − FORMULA − SAMPLE_ in 96% of the cases, and *P*_ERROR − EMPIRICAL_ > *P*_ERROR − FORMULA − OPTIMAL_ in 54% of the calculations. In the 4% of cases where *P*_ERROR − EMPIRICAL_ > *P*_ERROR − FORMULA − SAMPLE_, the two values differ by at most 8% and on average by 3%.

As previously observed, in research with real world data *P*_ERROR − FORMULA − OPTIMAL_ is inaccessible because it requires exact knowledge of the distribution. Real world judgments must be based on the relative magnitudes of *P*_ERROR − FORMULA − SAMPLE_ which uses values calculated from a finite sample drawn from the distribution. A simulation is not a theorem. Thus, simulations cannot provide a definitive determination, but with this limitation clearly in mind, the simulations suggest that in the case of a two dimensional classification *P*_ERROR − EMPIRICAL_, which is a more reliable estimate of classification error, will be greater than the more readily calculated *P*_ERROR − FORMULA − SAMPLE_.

### E. Coefficient of determination

Flury and Riedwyl (10).

$R_{\mathrm{A,B}}^{2}$ is the coefficient of determination between-Group A and Group B. It is the fraction of between-group variance that can be accounted for with these measures. As described in the text, it is used to select model variables in a backward elimination procedure.

$$R_{\mathrm{A,B}}^{2}=\frac{N_{A}N_{B}D_{\mathrm{A,B}}^{2}}{\left(N_{A}+N_{B}\right)\left(N_{A}+N_{B}-2\right)+N_{A}N_{B}D_{\mathrm{A,B}}^{2}}$$

where $D_{\mathrm{A,B}}^{2}$ is the between-group Mahalanobis distance.

### F. Sensitivity and specificity

Definitions of the standard measures of a diagnostic system follow. The notation follows that in Portney and Watkins (109).

*N* = Number of participants
A = Number of true positives
B = Number of false positives
C = Number of false negatives
D = Number of true negatives
Sensitivity = *A*/(*A* + *C*)
Specificity = *D*/(*B* + *D*)
Diagnostic accuracy = (*A* + *D*)/*N*
False positive Rate = *B*/(*B* + *D*)
False negative rate = *C*/(*A* + *C*)
Positive predictive value = *A*/(*A* + *B*)
Negative predictive value = *D*/(*C* + *D*)
Prevalence = (*A* + *C*)/*N*

### G. Calculation of single-variable effect size

The presentation here follows the detailed development in Ellis (110). Effect sizes are measures of the difference in a variable obtained in two groups normalized against a measure of the variable’s standard deviation. The three commonly used definitions differ in the specification of the normalization. Let ${\hat{\mu }}_{A}$ be the sample mean of the variable obtained from members of Group A. ${\hat{\sigma }}_{A}$ is the sample standard deviation of that mean. ${\hat{\mu }}_{B}$ and ${\hat{\sigma }}_{B}$ are defined analogously for Group B. *N*_A_ and *N*_B_ are the number of members in each group.

If the standard deviations of the two groups are approximately equal, then a pooled standard deviation is used to calculate Cohen’s *d* (111)

$$\begin{array}{llll} d & =\left({\hat{\mu }}_{A}-{\hat{\mu }}_{B}\right)∕{\hat{\sigma }}_{\mathrm{Pooled}}\; & & \;\\ {\hat{\sigma }}_{\mathrm{Pooled}} & =\left[\left(\overset{N_{A}}{\underset{m=1}{\sum }}{\left(x_{A}\left(m\right)-{\hat{\mu }}_{A}\right)}^{2}\right.\right.\; & & \;\\ & \;{\left.\left.+\overset{N_{B}}{\underset{m=1}{\sum }}{\left(x_{B}\left(m\right)-{\hat{\mu }}_{B}\right)}^{2}\right)∕\left(N_{A}+N_{B}-2\right)\;\right]}^{1∕2}\; & & \; \end{array}$$

A commonly used simplified version is

$${\hat{\sigma }}_{\mathrm{Pooled}}={\left\{\left({\hat{\sigma }}_{A}^{2}+{\hat{\sigma }}_{B}^{2}\right)∕2\right\}}^{1∕2}$$

In cases where the homogeneity of variance assumption is violated, Glass et al. (112) recommended using the standard deviation of the control group to compute Δ.

$$\Delta =\left({\hat{\mu }}_{A}-{\hat{\mu }}_{B}\right)∕{\hat{\sigma }}_{Control}$$

where ${\hat{\sigma }}_{Control}$ is either ${\hat{\sigma }}_{A}$ or ${\hat{\sigma }}_{B}$.

If the two groups are of significantly different sizes, then Hedges (113) recommended calculating the effect size using a weighted pooled standard deviation.

$$\begin{array}{c} g=({\hat{\mu }}_{\text{A}}-{\hat{\mu }}_{\text{B}})/{\hat{\sigma }}_{\text{Weighted}}\\ {\hat{\sigma }}_{\text{Weighted}}={\left\{\frac{(N_{\text{A}}-1)\;{\hat{\sigma }}_{\text{A}}^{2}+(N_{\text{B}}-1){\hat{\sigma }}_{\text{B}}^{2}}{N_{\text{A}}+B_{\text{B}}-2}\right\}}^{1/2} \end{array}$$

Hedges’ *g* is seen to be the special case of the between-group Mahalanobis distance for the *Z* = 1 case. If *N*_A_ = *N*_B_, which is the case for treatment studies where all participants complete the trial, then Hedges’ *g* is the same as Cohen’s *d*.
